# PrEP choice in the real world: Results of a prospective cohort study describing uptake and use patterns of oral PrEP and the dapivirine vaginal ring among women in sub‐Saharan Africa

**DOI:** 10.1002/jia2.26457

**Published:** 2025-07-02

**Authors:** Virginia A. Fonner, Elizabeth Irungu, Mark Conlon, Carolyne A. Akello, Emily Gwavava, Kevin K'Orimba, Nicolette P. Naidoo, Patriciah Jeckonia, Imelda Mahaka, Saiqa Mullick, Mamatli Chabela, Roisin Drysdale, Jacqueline Kabongo, Millicent Kiruki, Ivan Segawa, Munyaradzi Dobbie, Nthuseng Marake, Peter Mudiope, Hasina Subedar, Rose Wafula, Andrew Kazibwe, Jason Reed, Katharine Kripke, Douglas Taylor, Mu‐Tien Lee, Glory Chidumwa, Adatia Chivafa, Ramatsoai Soothoane, Margaret Eichleay, Ashley Mayo, Courtney McGuire, Tara McClure, Tatenda Yemeke, Kristine Torjesen, the CATALYST study team

**Affiliations:** ^1^ FHI 360 Durham North Carolina USA; ^2^ Jhpiego Nairobi Kenya; ^3^ FHI 360 Kampala Uganda; ^4^ Pangaea Zimbabwe Harare Zimbabwe; ^5^ LVCT Health Nairobi Kenya; ^6^ Wits RHI, University of the Witwatersrand Johannesburg South Africa; ^7^ Zimbabwe Ministry of Health Harare Zimbabwe; ^8^ Lesotho Ministry of Health Maseru Lesotho; ^9^ Uganda Ministry of Health Kampala Uganda; ^10^ National Department of Health Pretoria South Africa; ^11^ Kenya Ministry of Health Nairobi Kenya; ^12^ TASO Kampala Uganda; ^13^ Jhpiego, Baltimore Maryland USA; ^14^ Avenir Health Takoma Park Maryland USA; ^15^ School of Public Health, University of the Witwatersrand Johannesburg South Africa

**Keywords:** female, Kenya, Lesotho, pre‐exposure prophylaxis, South Africa, Uganda, Zimbabwe

## Abstract

**Introduction:**

HIV incidence remains high among women in Africa, especially adolescent girls and young women (AGYW), despite existing oral pre‐exposure prophylaxis (PrEP) programmes. With expanding biomedical prevention options, understanding PrEP use patterns when women are offered choice can inform HIV prevention programming in Africa.

**Methods:**

The CATALYST study offers informed PrEP choice through an enhanced service delivery package for women in 27 public health sites across Kenya, Lesotho, South Africa, Uganda, and Zimbabwe. Women attending sites who were HIV negative and interested in learning about HIV prevention were eligible. We describe uptake and use among those offered choice between oral PrEP and the monthly dapivirine ring from May 2023 through July 2024, explore factors associated with method choice using logistic regression, describe reasons for choice and assess time until PrEP discontinuation using survival analysis.

**Results:**

Of 3967 participants, 44.9% were AGYW (15−24 years), 25.5% were sex workers, and 12.2% and 8.7% were breastfeeding and/or pregnant, respectively. At enrolment, 66.2% chose oral PrEP, 29.9% chose the dapivirine ring and 3.5% chose no method. Common reasons for choosing oral PrEP were ease of use (58.6%) and efficacy (31.7%); the ring was chosen due to ease of use (56.9%) and not needing to swallow pills (53.0%). In multivariable analysis, participants ≤ 24 years (*p* = 0.007) and participants who were pregnant (*p* = 0.002) or breastfeeding (*p* < 0.001) had lower odds of choosing the ring. Month 1 return was 32.7% for oral PrEP and 55.2% for the ring. Ring users reported higher adherence as compared to oral PrEP users (*p* < 0.001). Of participants returning for ≥ 1 PrEP refills, 12.1% switched methods at least once. Median time until PrEP discontinuation was 95 days (95% CI: 91, 110) for those choosing oral PrEP at enrolment and 169 days (95% CI: 139, 190) for those choosing the ring. Risk of discontinuation was greater for participants choosing oral PrEP at enrolment (*p* < 0.001) and those ≤ 24 years (*p* < 0.001), PrEP naïve at enrolment (*p* < 0.001) or not currently using contraception (*p* = 0.03).

**Conclusions:**

We demonstrated that women took advantage of PrEP choice. PrEP use varied by product, with 1 month return and method continuation higher for the ring. AGYW had a greater risk of discontinuing either method, suggesting more support is needed.

## 1 INTRODUCTION

In 2023, an estimated 1.3 million individuals acquired HIV globally, with half occurring in Africa. Women and girls in sub‐Saharan Africa (SSA) are disproportionately affected, accounting for an estimated 62% of all new regional acquisitions [1, 2]. Addressing high rates of HIV transmission among women in SSA remains a critical priority. The introduction and scale‐up of effective HIV prevention interventions is urgently needed.

In 2015, the World Health Organization (WHO) recommended the use of daily oral pre‐exposure prophylaxis (PrEP) containing tenofovir as PrEP [3]. Oral PrEP is highly effective at reducing HIV‐1 acquisition when used as directed. Most countries in SSA have incorporated oral PrEP into their HIV prevention programmes, with more than 5 million individuals initiated regionally [4]. However, product‐related, individual, social and structural barriers have resulted in suboptimal PrEP use [5]. While some studies have demonstrated relatively high rates of oral PrEP uptake among women and adolescent girls, early discontinuation and low adherence are common [6, 7, 8].

Newer PrEP formulations that are discreet and long‐acting may address some barriers and improve use [9]. The dapivirine ring, a silicone ring inserted vaginally every month, is a discreet PrEP formulation that does not require daily dosing, has few side effects and is accepted by women who would benefit from HIV prevention [10]. When used as directed, the dapivirine ring is moderately effective at reducing rates of HIV‐1 acquisition from vaginal sexual exposure [11, 12]. In 2021, WHO recommended the monthly dapivirine ring for women as an additional PrEP option [13]. The dapivirine ring is approved for use in several African countries [14]. WHO has also recommended long‐acting injectable cabotegravir (CAB PrEP), a highly effective PrEP formulation requiring dosing every 2 months [15, 16].

Offering multiple HIV prevention options can enhance prevention coverage and reduce HIV incidence because individuals may select the product most aligned with their unique needs and preferences [17, 18]. Healthcare providers need to support informed decision‐making by educating potential users about all available HIV prevention options [19]. As new HIV prevention options—such as the dapivirine ring and CAB PrEP—are introduced, evidence is limited on PrEP choice implementation in public health settings [20, 21]. Additionally, data on use patterns, including transitions on/off PrEP and switching between products, remain scarce [22]. We are conducting an implementation science study to provide real‐world evidence on delivering informed PrEP choice for women within existing PrEP programmes in SSA.

## 2 METHODS

### 2.1 Study design, setting and population

CATALYST is a prospective cohort study and mixed‐methods process evaluation. The study includes two distinct stages based on product approval and availability. Sites began Stage I once the dapivirine ring received regulatory approval and was available to participants (oral PrEP was already available). Countries transition to Stage II once CAB PrEP is approved and becomes available. Interim results described herein pertain only to Stage I and reflect data collected from May 2023 to July 2024 when CAB PrEP was not yet available.

Study sites in Stage I included 27 PEPFAR/USAID‐supported public health facilities across Kenya, Lesotho, South Africa, Uganda and Zimbabwe. To be eligible, women had to test HIV negative at enrolment, be interested in learning about HIV prevention, agree to study team contacts and meet age requirements. The study aimed to include individuals aged ≥ 15 years; age‐based eligibility among mature/emancipated minors varied by country (Appendix S1). “Women” were defined inclusively as individuals assigned female at birth of any gender identity or individuals assigned male at birth who identify as women. Pregnant and breastfeeding women could enrol, although their ability to choose between products varied by country.

### 2.2 Intervention

CATALYST offers PrEP choice by building on existing oral PrEP service delivery and in accordance with national guidelines. Enrolled participants are counselled on HIV prevention methods, including all available PrEP methods, and may choose their preferred method or switch methods at any visit, assuming they meet country‐specific product eligibility criteria (Appendix S2). Participants also receive pregnancy testing at each visit, subject to availability and clinical need, and are asked about their breastfeeding status. During periods of PrEP use, HIV testing is conducted per national testing algorithms and following national PrEP guidelines. Providers conduct informed choice counselling with participants. Training for counselling emphasized the importance of clients knowing all available methods, receiving method‐specific information in an understandable manner and being allowed to choose the method best suited for them. Providers were also given tools to use with clients, including method‐specific fact sheets and the HIV Prevention Journey Tool [23].

**Figure 1:**
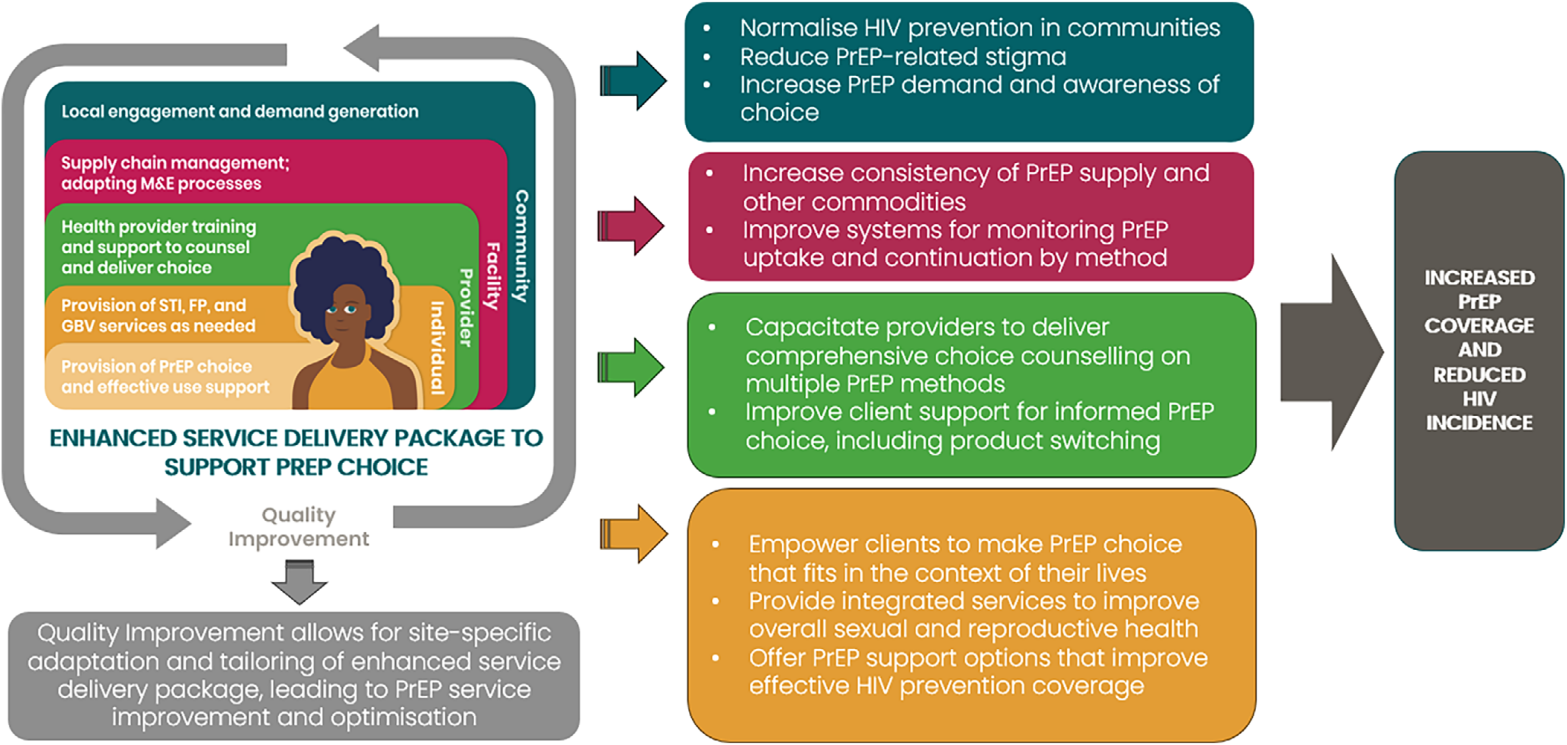
**The enhanced service delivery package for PrEP choice being implemented in CATALYST involved interventions at each level of the socio‐ecological model. Implementation of the package differed by site and could be adapted over the course of the study through site‐specific quality improvement initiatives**. Abbreviations: FP, family planning; GBV, gender‐based violence; M&E, monitoring and evaluation; PrEP, pre‐exposure prophylaxis; STI, sexually transmitted infection.

CATALYST implements an enhanced service delivery package with components at the individual, provider, facility and community levels (Figure 1). This package is expected to work through multiple pathways, ultimately leading to improved PrEP coverage and decreased HIV incidence. As an implementation strategy allowing for intervention adaptation, the study supports existing quality improvement mechanisms in each county/district and site to enable site‐level refinements.

### 2.3 Data collection procedures and outcomes

Participants were administered structured questionnaires at enrolment and each subsequent PrEP‐related clinic visit. Visits were aligned with dosing schedules per national PrEP guidelines. For those who missed a PrEP‐related visit, study staff attempted to contact participants 1 and 3 months later to administer a brief questionnaire related to PrEP use via phone or other preferred means. Unless participants expressly stated their desire to withdraw from the study, participants remained in CATALYST even if they stopped PrEP clinic visits and could return at any point to continue/restart PrEP. Participants who tested HIV positive were referred to existing HIV treatment services and exited from the study.

At baseline, data were collected on socio‐demographic characteristics and history of prior PrEP use. At each PrEP clinic visit, participants were asked about the method they chose and self‐reported method adherence, including any gaps in use or method cessation. We also reviewed visit records in which providers noted the PrEP method prescribed, amount provided and HIV testing results.

### 2.4 Analysis

To explore use patterns in Stage I, we assessed differences in method choice at enrolment using Chi‐squared tests of homogeneity and used logistic regression to assess socio‐demographic and behavioural factors associated with method choice. Factors associated with method choice (*p* < 0.05) were retained in a multivariable model.

Periods of PrEP use and non‐use were categorized using both PrEP visit data and self‐reported PrEP use and method adherence within the past 30 days, ascertained at clinic visits and during missed visit contacts. We analysed rates of initial refill and method switching descriptively. We assessed time until PrEP discontinuation of any method (excluding method switching), defined as the last time at which participants said they stopped using PrEP, or the time at which they would have run out of PrEP (without returning for a refill) without reporting use in the last 30 days at last study contact.

Factors associated with discontinuation were assessed using log‐rank tests and further explored using Cox proportional hazards regression models. Models included an interaction with time before or after 31 days to account for differences in the hazard ratio for variables in this initial study period. Participants who did not return at month 1 and were not reached after enrolment were assumed to have used their 30‐day supply, so day 31 was assumed to be when these participants ran out (and discontinued use). We also calculated the total volume of PrEP dispensed by method and assessed HIV seroconversions occurring during the analysis period.

Results are based on participants who enrolled into Stage I only. Because some participants may return during Stage II, final Stage I results (available following Stage II completion) could change slightly. Analyses were conducted across all countries, although when feasible country was included as a factor to assess between‐country differences. Analyses were conducted using SAS (v8.2) and Stata 18.

### 2.5 Ethical considerations

CATALYST received ethical approval from the following institutional review boards: Kenya Medical Research Institute Scientific and Ethics Review Unit and the National Commission for Science, Technology and Innovation; Lesotho National Health and Research Ethics Committee; Johns Hopkins Bloomberg School of Public Health; University of the Witwatersrand's Human Research Ethics Committee; University of the Free State Health Sciences Research Ethics Committee; South African National Health Research Database; National HIV/AIDS Research Committee of Uganda; Uganda National Council for Science and Technology; and the Medical Research Council of Zimbabwe. Participants provided written informed consent. Participants ages 15–17 were eligible if they met country‐specific definitions of mature/emancipated minors. Participants were not provided remuneration for attending regularly scheduled clinic visits but were offered refreshments during the completion of study‐related questionnaires.

## 3 RESULTS

### 3.1 Study population

**Figure 2:**
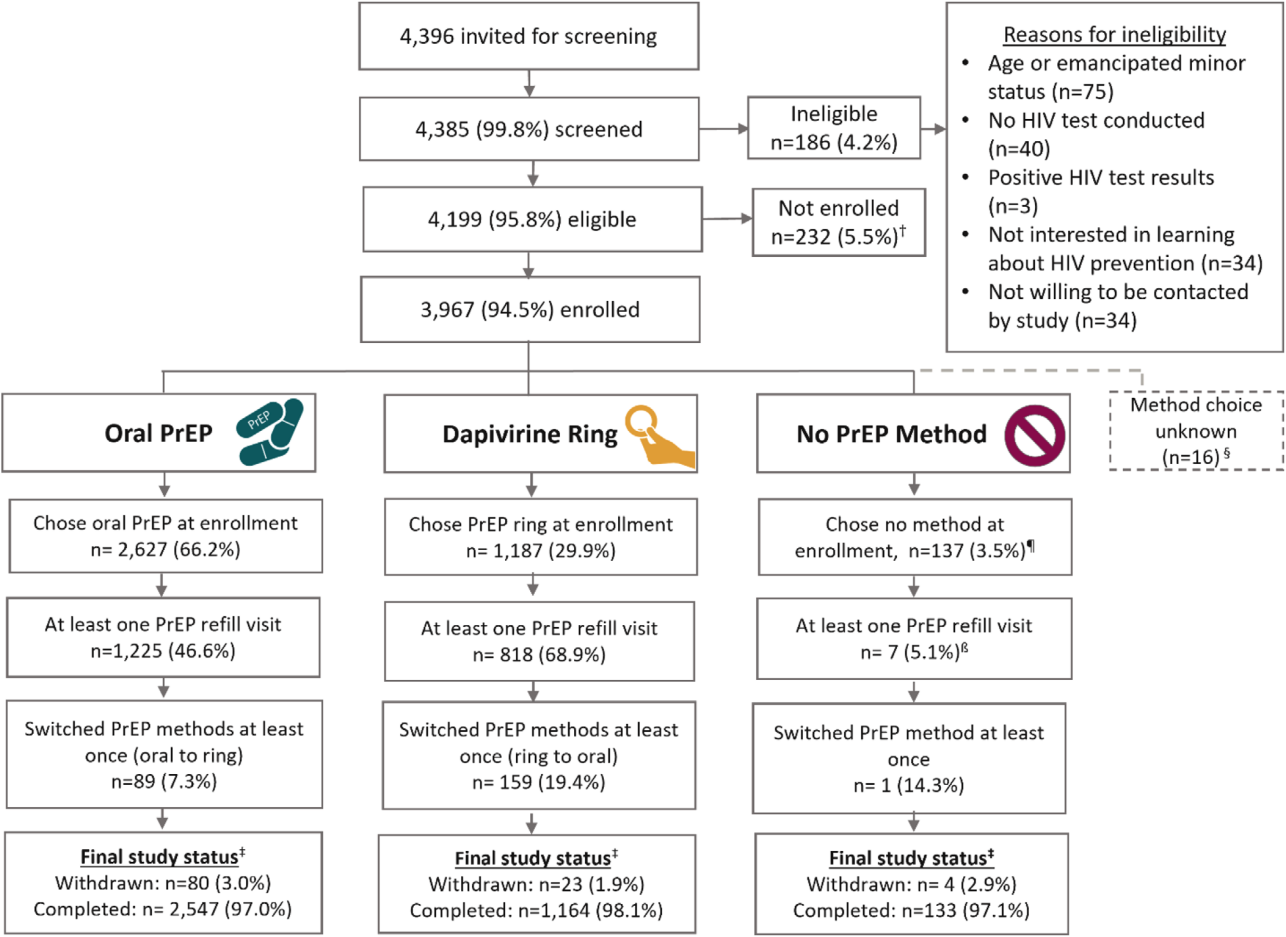
**Disposition of CATALYST participants in Stage I of the study**. Abbreviation: PrEP, pre‐exposure prophylaxis. ^†^Reasons for not enrolling following confirmation of eligibility included not having time to complete study procedures and declining enrollment during the informed consent process. ^‡^For final study status, “Completed” refers to any participant who did not withdraw from the study, even if they had no recent contact with the site or study staff. ^§^Method selection is unknown for 5 participants, and an additional 11 participants completed eligibility screening and enrolment but had no further data collected; hence, their method choice is also listed as unknown. ^β^ This cell includes individuals who returned to initiate PrEP after enrolment and returned for a PrEP refill at least once. ^¶^An additional nine people who chose no method at enrolment eventually returned to initiate PrEP but never returned for a refill.

Of 4396 women invited for eligibility screening, 3967 were enrolled in Stage I (Figure 2). Across countries, 44.9% of participants were adolescent girls and young women (AGYW) aged 15–24 years, 25.5% were sex workers, and 12.2% and 8.7% were breastfeeding and/or pregnant, respectively (Table 1; categories are not mutually exclusive). Since country guidelines differed regarding eligibility for the ring based on age, pregnancy and/or breastfeeding status, 91.0% of participants could choose between PrEP products; the remainder were eligible only for oral PrEP. Regarding prior PrEP use at enrolment, 67.5% were PrEP naïve, 16.6% reported currently using oral PrEP and 14.7% had previously used oral PrEP but not within 30 days of enrolment. About a third of participants reported having multiple sexual partners in the past 3 months, and about 50% reported engaging in condomless vaginal sex in the past month.

**Table 1:** Stage I CATALYST participant characteristics at enrolment

	Kenya	Lesotho	South Africa	Uganda	Zimbabwe	Total
*Total N*	766	872	557	837	935	3967
***Socio‐demographic characteristics***						
**Age** (mean in years, SD)	27.3 (SD 7.4)	25.4 (SD 6.9)	29.9 (SD 8.0)	26.8 (SD 7.4)	29.2 (SD 8.2)	27.6 (SD 7.7)
**Prior PrEP use** ^a^, *n* (%)						
PrEP naïve	433 (56.5)	676 (77.5)	272 (48.8)	645 (77.1)	651 (69.6)	2677 (67.5)
Former PrEP user	172 (22.5)	99 (11.4)	121 (21.7)	104 (12.4)	88 (9.4)	584 (14.7)
Current PrEP user	144 (18.8)	90 (10.3)	161 (28.9)	81 (9.7)	183 (19.6)	659 (16.6)
**Marital status**, *n* (%)						
Never married	395 (51.6)	428 (49.1)	468 (84.0)	340 (40.6)	170 (18.2)	1801 (45.4)
Married	218 (28.5)	394 (45.2)	52 (9.3)	334 (39.9)	512 (54.8)	1510 (38.1)
Divorced, widowed, separated	134 (17.5)	42 (4.8)	34 (6.1)	153 (18.3)	239 (25.6)	602 (15.2)
**Education**, *n* (%)						
Less than completed primary	89 (11.6)	8 (0.9)	7 (1.3)	295 (35.2)	29 (3.1)	428 (10.8)
Completed primary	136 (17.8)	59 (6.8)	14 (2.5)	142 (17)	98 (10.5)	449 (11.3)
Some secondary	131 (17.1)	288 (33.0)	209 (37.5)	300 (35.8)	675 (72.2)	1603 (40.4)
Completed secondary	227 (29.6)	293 (33.6)	268 (48.1)	8 (1.0)	48 (5.1)	844 (21.3)
More than secondary	165 (21.5)	217 (24.9)	56 (10.1)	85 (10.2)	73 (7.8)	596 (15.0)
**Time to facility**, *n* (%)						
< 30 minutes	467 (61.0)	458 (52.5)	450 (80.8)	619 (74.0)	629 (67.3)	2623 (66.1)
30 minutes−1 hour	208 (27.2)	261 (29.9)	89 (16.0)	135 (16.1)	196 (21.0)	889 (22.4)
> 1 hour	71 (9.3)	115 (13.2)	13 (2.3)	65 (7.8)	92 (9.8)	356 (9.0)
**Risk perception (worry about acquiring HIV)**, *n* (%)						
Not worried	632 (82.5)	686 (78.7)	456 (81.9)	597 (71.3)	526 (56.3)	2897 (73.0)
Some worry	84 (11.0)	128 (14.7)	60 (10.8)	168 (20.1)	214 (22.9)	654 (16.5)
Worry a lot	26 (3.4)	39 (4.5)	33 (5.9)	63 (7.5)	147 (15.7)	308 (7.8)
**Currently in school**, *n* (%)						
Yes	97 (12.7)	177 (20.3)	86 (15.4)	77 (9.2)	56 (6.0)	493 (12.4)
No	652 (85.1)	684 (78.4)	467 (83.8)	752 (89.8)	863 (92.3)	3418 (86.2)
**Primary partner**, *n* (%)						
Yes	615 (80.3)	819 (93.9)	435 (78.1)	730 (87.2)	839 (89.7)	3438 (86.7)
No	133 (17.4)	45 (5.2)	120 (21.5)	100 (11.9)	84 (9.0)	482 (12.2)
***Population***						
**AGYW (15**−**24 years)** ^b^, *n* (%)						
Yes	349 (45.6)	525 (60.2)	169 (30.3)	404 (48.3)	333 (35.6)	1780 (44.9)
No	417 (54.4)	347 (39.8)	388 (69.7)	433 (51.7)	602 (64.4)	2187 (55.1)
**Pregnant at enrolment**, *n* (%)						
Yes	54 (7.0)	121 (13.9)	14 (2.5)	49 (5.9)	109 (11.7)	347 (8.7)
No	694 (90.6)	734 (84.2)	540 (96.9)	781 (93.3)	821 (87.8)	3570 (90)
**Breastfeeding at enrolment**, *n* (%)						
Yes	137 (17.9)	84 (9.6)	28 (5.0)	86 (10.3)	147 (15.7)	482 (12.2)
No	611 (79.8)	781 (89.6)	528 (94.8)	744 (88.9)	776 (83)	3440 (86.7)
**Sex worker** ^c^, *n* (%)						
Yes	357 (46.6)	48 (5.5)	233 (41.8)	245 (29.3)	127 (13.6)	1010 (25.5)
No	393 (51.3)	820 (94)	321 (57.6)	583 (69.7)	798 (85.3)	2915 (73.5)
**Transgender**						
Yes	3 (0.4)	2 (0.2)	2 (0.4)	0 (0.0)	2 (0.2)	9 (0.2)
No	763 (99.6)	870 (99.8)	555 (99.6)	0 (0.0)	778 (83.2)	2966 (74.8)
***Behaviours***						
**Currently using a contraceptive method**, *n* (%)						
Yes	455 (59.4)	441 (50.6)	370 (66.4)	348 (41.6)	546 (58.4)	2160 (54.4)
No	238 (31.1)	299 (34.3)	167 (30)	434 (51.9)	268 (28.7)	1406 (35.4)
Currently pregnant	54 (7)	121 (13.9)	14 (2.5)	49 (5.9)	109 (11.7)	347 (8.7)
**Sexual partners in the past 3 months**, *n* (%)						
0−1 partner	380 (49.6)	726 (83.3)	347 (62.3)	529 (63.2)	737 (78.8)	2719 (68.5)
>1 partners	357 (46.6)	137 (15.7)	201 (36.1)	297 (35.5)	186 (19.9)	1178 (29.7)
**Any condomless vaginal sex in the past month,** *n* (%)						
No condomless sex	310 (40.5)	358 (41.1)	241 (43.3)	247 (29.5)	360 (38.5)	1516 (38.2)
Any condomless sex	400 (52.2)	486 (55.7)	228 (40.9)	570 (68.1)	551 (58.9)	2235 (56.3)
**Ability to choose between PrEP products** ^d^, *n* (%)						
Yes	760 (99.2)	847 (97.1)	509 (91.4)	680 (81.2)	813 (87.0)	3609 (91.0)
No	6 (0.8)	25 (2.9)	44 (7.9)	150 (17.9)	117 (12.5)	342 (8.6)

***Note about missing data***: Some data were missing for most variables (<3%); column percentages account for missing. Missing data were higher than 3% for some variables, including time to facility in Lesotho (4.4% missing), risk perception (3.1% missing in Kenya; 5.1% missing in Zimbabwe), transgender status (100% missing in Uganda; 16.6% missing in Zimbabwe), number of sex partners in Kenya (3.8%) and condomless sex (7.3% in Kenya; 15.8% in South Africa).

***Note about participant profile differences across countries***: All variables in table differed across countries (*p*<0.001). Categorical variables and continuous variables that have been categorized at discrete levels were tested using Cochran−Mantel−Haenszel test. Continuous variables were analysed by Wilcoxon Mann−Whitney test.

Abbreviations: AGYW, adolescent girls and young women; PrEP, pre‐exposure prophylaxis; SD, standard deviation.

^a^ “PrEP naïve” was defined as never having used PrEP prior to study enrolment. “Current” PrEP user was defined as reporting previously using a PrEP method within the last 30 days of enrolment. “Former” PrEP user was defined as previously having used a PrEP method prior to study enrolment but not within 30 days of joining the study.

^b^ Total of AGYW includes participants ages 15−17 years (*n* = 57), including 6 in Kenya, 24 in Lesotho, 0 in South Africa (where eligibility criterion was 18 years and older), 18 in Uganda and 9 in Zimbabwe.

^c^ Sex worker status was defined only for participants ages 18 years and above. Sex worker status was defined either by self‐report and/or through clinic record documentation of sex worker status.

^d^ Ability to choose between PrEP products was based on country‐specific product eligibility requirements, including age, pregnancy and breastfeeding status.

Median time in Stage I was 253 days (interquartile range [IQR]: 188, 310). Overall, 2.7% (*n* = 107 participants) withdrew from the study. Overall, about half of the participants returned to refill PrEP following enrolment, including 46.6% who selected oral PrEP and 68.9% who selected ring (Figure 2).

### 3.2 PrEP method choice

Of participants who chose a method at enrolment, 66.2% chose oral PrEP, 29.9% chose the dapivirine ring and 3.5% chose no PrEP method (Figure 2). Among PrEP‐naïve participants who had method choice at enrolment (*n* = 2312), there was a significant difference between choosing oral PrEP (*n* = 1686; 72.9%) versus the ring (*n* = 626; 27.1%) (*p* < 0.001). Being “easy to use” was the most common reason for choosing either method, reported by 58.6% of oral PrEP users and 56.9% of ring users. For oral PrEP, other common reasons included efficacy (31.7%) and no need for insertion (11.3%). For ring users, other reasons were not needing to swallow pills (53.0%), dosing schedule (18.4%) and ability to use discreetly (14.4%) (data not shown).

Method choice varied among countries. Lesotho had the lowest proportion of clients choosing ring (20.3%), whereas Kenya had the highest (45.1%), among participants who chose a method at enrolment and had choice (Table 2). Across countries, of participants who reported current PrEP use at enrolment (*n* = 630), 44.0% switched to ring, 54.6% remained on oral PrEP and 1.4% chose no method.

**Table 2:** Factors associated with choosing the dapivirine ring (versus oral PrEP) among participants who chose a PrEP method at enrolment and had a choice between methods

Participant characteristics^a^	Oral PrEP	Dapivirine ring	Total	Unadjusted OR (95% CI)	*p*‐value	Adjusted OR (95% CI)	*p*‐value
**Overall PrEP choice** ^b^	2301 (66.1)	1182 (33.9)	3483				
**Country**							
Kenya	400 (54.9)	329 (45.1)	729 (20.9)	3.23 (2.577, 4.037)	<.001	2.43 (1.866, 3.157)	<.001
Lesotho	651 (79.7)	166 (20.3)	817 (23.5)	reference	_		
South Africa	346 (69.6)	151 (30.4)	497 (14.3)	1.71 (1.325, 2.211)	<.001	0.99 (0.729, 1.347)	0.952
Uganda	377 (57.6)	278 (42.4)	655 (18.8)	2.89 (2.297, 3.641)	<.001	2.36 (1.796, 3.092)	<.001
Zimbabwe	527 (67.1)	258 (32.9)	785 (22.5)	1.92 (1.531, 2.408)	<.001	1.58 (1.222, 2.036)	<.001
Total	2301	1182	3483				
**Age**							
18−24	1240 (62.6)	742 (37.4)	1982 (56.9)	reference	_		
25+	1061 (70.7)	440 (29.3)	1501 (43.1)	0.69 (0.600, 0.800)	<.001	0.79 (0.658, 0.937)	0.007
Total	2301	1182	3483				
**Prior PrEP status at enrolment**							
PrEP naïve	1686 (72.9)	626 (27.1)	2312 (66.7)	0.46 (0.384, 0.554)	<.001	0.46 (0.373, 0.567)	<.001
Former PrEP user	258 (48.6)	273 (51.4)	531 (15.3)	1.31 (1.041, 1.658)	0.021	1.13 (0.871, 1.462)	0.360
Current PrEP user	344 (55.4)	277 (44.6)	621 (17.9)	reference	_		
Total	2288	1176	3464				
**Sex worker status**							
Sex worker	520 (53.6)	450 (46.4)	970 (28)	2.11 (1.810, 2.458)	<.001	1.05 (0.832, 1.320)	0.691
Non‐sex worker	1772 (70.9)	727 (29.1)	2499 (72)	reference	_		
Total	2292	1177	3469				
**Education**							
Up to completed primary	455 (60.5)	297 (39.5)	752 (21.7)	reference	_		
More than completed primary	1834 (67.6)	878 (32.4)	2712 (78.3)	0.73 (0.621, 0.867)	<.001	1.13 (0.917, 1.390)	0.252
Total	2289	1175	3464				
**Number of sexual partners in the past 3 months**							
0−1	1681 (72.1)	650 (27.9)	2331 (67.7)	reference	_		
>1	593 (53.4)	517 (46.6)	1110 (32.3)	2.25 (1.943, 2.616)	<.001	1.50 (1.218, 1.854)	<.001
Total	2274	1167	3441				
**Current contraceptive use**							
Yes	1273 (63.1)	743 (36.9)	2016 (58.4)	1.38 (1.192, 1.592)	<.001	1.27 (1.077, 1.502)	0.005
No	1010 (70.2)	428 (29.8)	1438 (41.6)	reference	_		
Total	2283	1171	3454				
**Any condomless vaginal sex in the past month**							
No condomless sex	952 (69.3)	421 (30.7)	1373 (41.5)	reference	_		
Any condomless sex	1227 (63.5)	705 (36.5)	1932 (58.5)	1.30 (1.121, 1.506)	<.001	1.33 (1.132, 1.575)	<.001
Total	2179	1126	3305				
**Worry about HIV**							
Not worried	1705 (66.1)	875 (33.9)	2580 (75.3)	reference	_		
Worried	553 (65.4)	293 (34.6)	846 (24.7)	1.03 (0.877, 1.216)	0.701		
Total	2258	1168	3426				
**Has primary partner**							
Yes	267 (60.3)	176 (39.7)	443 (12.8)	reference	_		
No	2021 (66.9)	1000 (33.1)	3021 (87.2)	0.75 (0.612, 0.921)	0.006	1.00 (0.783, 1.280)	0.993
Total	2288	1176	3464				
**Marital status**							
Never married	1083 (65.9)	561 (34.1)	1644 (47.6)	reference	_		
Married/living as married	899 (71.9)	351 (28.1)	1250 (36.2)	0.75 (0.642, 0.885)	<.001	0.80 (0.646, 0.989)	0.039
Divorced/separated/ widowed	304 (54)	259 (46)	563 (16.3)	1.64 (1.354, 1.998)	<.001	1.15 (0.906, 1.465)	0.247
Total	2286	1171	3457				
**Pregnancy status at enrolment**							
Pregnant	2158 (65.2)	1154 (34.8)	3312 (95.7)	reference	_		
Non‐pregnant	127 (85.2)	22 (14.8)	149 (4.3)	0.32 (0.205, 0.512)	<.001	0.41 (0.239, 0.719)	0.002
Total	2285	1176	3461				
**Breastfeeding status at enrolment**							
Breastfeeding	2025 (64.7)	1107 (35.3)	3132 (90.3)	reference	_		
Non‐breastfeeding	266 (79.4)	69 (20.6)	335 (9.7)	0.47 (0.361, 0.625)	<.001	0.46 (0.337, 0.629)	<.001
Total	2291	1176	3467				

Abbreviations: PrEP, pre‐exposure prophylaxis; OR, odds ratio; CI, confidence interval.

^a^ Any participant who had a choice among PrEP products was included in the model. Participants ineligible for the dapivirine ring based on age, pregnancy or breastfeeding status according to country guidelines were excluded. Notably, there could have been clinically relevant factors that prevented some additional participants from choosing between both methods (e.g. product stock‐out, creatinine levels), but these are not accounted for when defining the population who had choice. In Kenya and Lesotho, dapivirine ring use is allowed during pregnancy and breastfeeding. In Kenya, Lesotho and Zimbabwe, the use of PrEP ring is allowed during breastfeeding (following the post‐partum period). In all CATALYST countries, dapivirine ring use is not permitted for people aged below 18 years.

^b^ Sex worker status was defined only for participants ages 18 years and above. Sex worker status was defined either by self‐report and/or through clinic record documentation of sex worker status.

In multivariable analysis, being ≤ 24 years (vs. > 24 years) was associated with lower odds of choosing ring (aOR = 0.79 [95% CI: 0.658, 0.937], *p* = 0.007) (Table 2). PrEP‐naïve participants had lower odds of choosing ring compared to those using oral PrEP at enrolment (aOR = 0.46 [95% CI: 0.373, 0.567], *p* < 0.001). Women using a contraceptive method were more likely to choose the ring compared to those not using contraception (aOR = 1.27 [95% CI: 1.077, 1.502], *p* = 0.005). Having > 1 recent sexual partners was associated with an increased likelihood of choosing ring (aOR = 1.50, [95% CI: 1.218, 1.854], *p* < 0.001), as was reporting recent condomless sex (aOR = 1.33 [95% CI: 1.123, 1.575], *p* < 0.001). Sex workers had an increased likelihood of choosing ring, but this association was not significant in multivariable analyses controlling for potentially confounding variables, including age (Table 2).

In the all‐country model, women who had choice and were pregnant and/or breastfeeding at enrolment had lower odds of choosing the ring compared to non‐pregnant and non‐breastfeeding women. Findings in models restricted to countries allowing choice among pregnant women (Kenya and Lesotho) and among breastfeeding women (Kenya, Lesotho and Zimbabwe) demonstrated similar results.

### 3.3 PrEP use, product adherence and method switching

**Figure 3:**
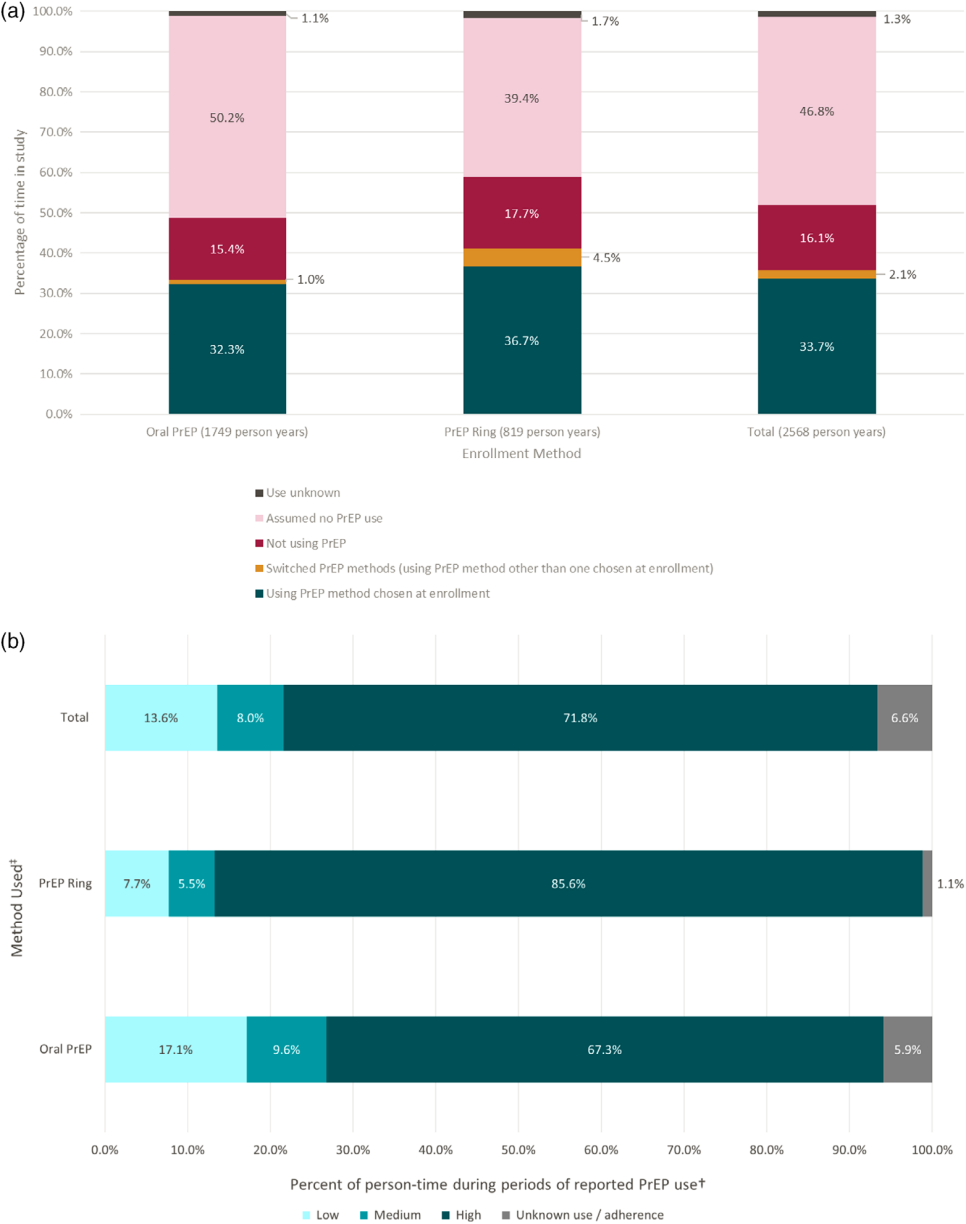
**(a) Periods of PrEP use and non‐use during Stage I of CATALYST**. Periods of PrEP use and non‐use were defined by participant responses to questions related to PrEP use within the past 30‐days, asked either at a clinic visit related to PrEP or during a missed visit call. If someone missed a visit but reported continued PrEP use, this time was considered time using PrEP, as someone could either have acquired oral PrEP from a non‐CATALYST site or could have been maintaining less than high adherence, either of which could explain the missed visit. Time not using PrEP reflects time in which a participant reported no PrEP use. Assumed time not using PrEP includes time during which a participant remained in CATALYST but was unable to be reached for missed visit phone calls, or following the time of the last study contact (∼3 months following a missed visit) in which a participant technically remained in CATALYST but did not return to a CATALYST site for additional PrEP. Unknown time reflects time in which PrEP use was unaccounted for; this time was excluded from calculating the overall percentages. **(b) Self‐reported adherence to the PrEP method during periods of use**. ^†^Self‐reported adherence was categorized as high, medium, and low, with “high” adherence defined as reportedly using PrEP within the last ≥ 29/30 days with no gaps of > 1 day in time since last visit. “Medium” adherence was defined as use 60% of the time within the past 30 days (≥ 18 days), with no gaps in use of > 4 days since last visit, and “low” adherence as some use of the method, but insufficient to meet the definition of medium adherence. ^‡^Method use refers to any time in which a particular PrEP method was reportedly used. If someone switched PrEP methods during the study, their self‐reported adherence would be split by method. Abbreviation: PrEP, pre‐exposure prophylaxis.

Of 2568 total person years among those choosing a method at enrolment, 33.7% was time in which participants reported using the PrEP method initially chosen, 2.1% was time in which a different method was used (i.e. method switching) and 16.1% was time in which participants reported not using PrEP. Most remaining time (46.8%) comprised periods when participants were assumed not to be using PrEP due to not returning for additional visits (Figure 3a). During periods of PrEP use, participants commonly reported high adherence, defined as using PrEP within the last ≥ 29/30 days with no gaps of > 1 day since the last contact (67.3% of time for oral PrEP users and 85.6% of time for ring users). Ring users had significantly more person‐time classified as high adherence (vs. less‐than‐high adherence) compared to oral PrEP users (*p* < 0.001) (Figure 3b).

Among those with a scheduled 1‐month return visit (*n* = 3509), 34.4% of oral PrEP users returned at month 1 (allowing for a 30‐day grace period, i.e. within 60 days of product receipt), including 32.7% who refilled oral PrEP and 1.8% who switched methods. Among ring users, 61.4% returned, including 55.2% who refilled rings and 6.2% who switched methods. However, among those who did not refill and were reached, 21.9% (*n* = 163/743) reported continued PrEP use during their missed visit contact, including 24.9% (*n* = 145/582) for oral PrEP and 11.2% (*n* = 18/161) for ring. Factors associated with refill at month 1 are reported in Appendix S3.

Of participants with ≥ 1 refill visit who chose a method at enrolment, 12.1% (*n* = 248/2050) switched methods at least once, with 7.3% (*n* = 89/1225) switching from oral PrEP to the ring and 19.4% (*n* = 159/818) from the ring to oral PrEP (*p* < 0.001). Median time until the first switch was 43 days (IQR: 29, 104) among those who switched to the ring and 61 days (IQR: 29, 128) among those who switched to oral PrEP. Thirty participants switched products more than once.

### 3.4 Time until PrEP discontinuation

**Figure 4:**
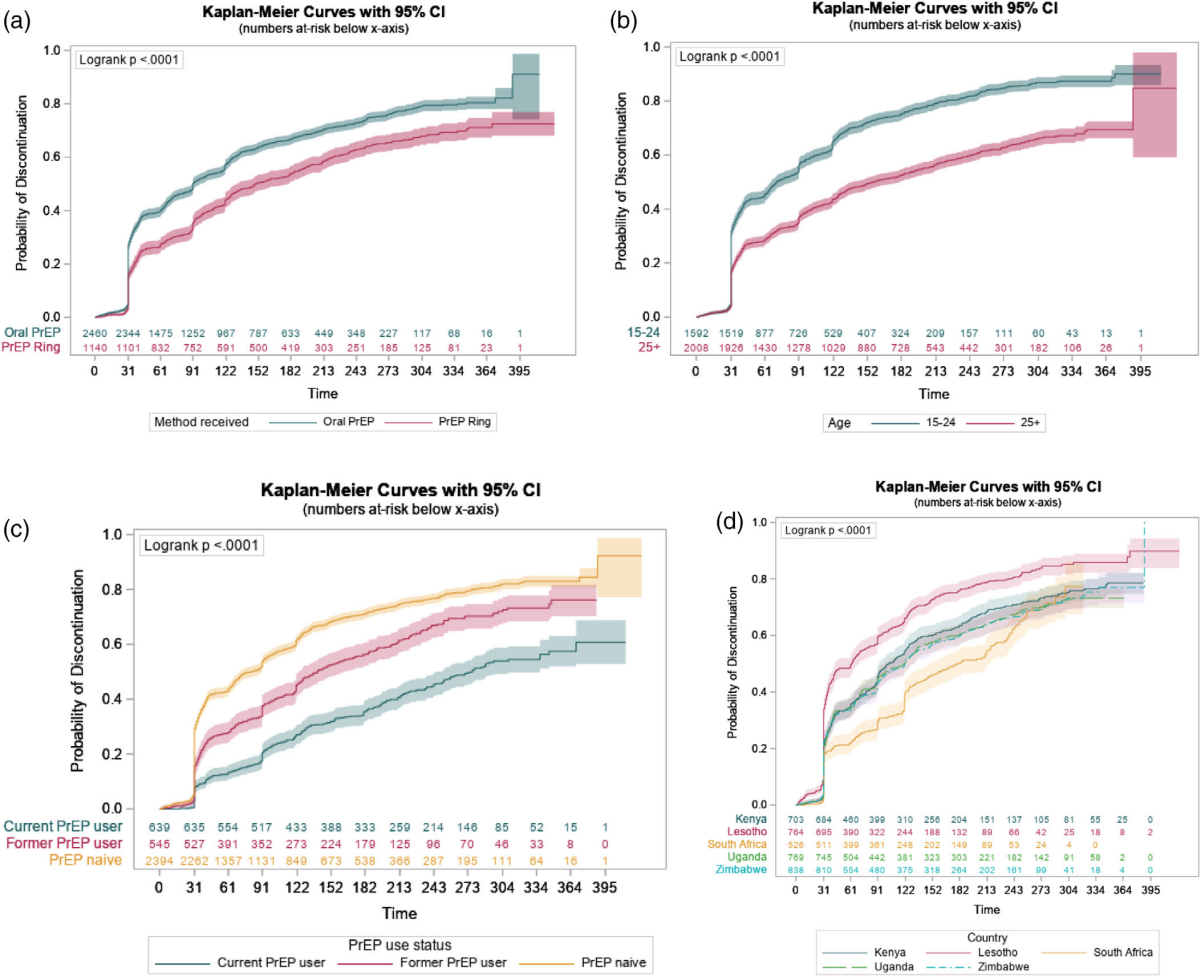
**Time until PrEP discontinuation by (a) PrEP method, (b) age, (c) PrEP experience at enrolment and (d) country**. Abbreviations: CI, confidence interval; PrEP, pre‐exposure prophylaxis.

Median time until PrEP discontinuation was 91 days (95% CI: 91, 98) for those selecting oral PrEP at enrolment, compared to 149 days (95% CI: 132, 180) for those selecting ring (log‐rank *p* < 0.001; Table 3 and Figure 4). Time until discontinuation varied by country (*p* < 0.001), with the lowest median time in Lesotho (62 days) and the highest in South Africa (181 days). Other baseline factors significantly associated with higher rates of discontinuation based on log‐rank tests included age ≤ 24 years; never married; being PrEP naïve; currently in school; and having more than a primary school education. Baseline factors associated with reduced risk of discontinuation included being a sex worker and current contraception use. Being pregnant or breastfeeding at enrolment was not associated with risk of discontinuation compared to non‐pregnant (*p* = 0.224) and non‐breastfeeding participants (*p* = 0.211), respectively.

**Table 3:** Factors associated with PrEP discontinuation among participants who chose a PrEP method at enrolment during Stage I

Participant characteristics ^a^	Median survival days (95% CI)	log rank *p*‐value (overall)	Hazard ratio before day 31 (95% CI)	Hazard ratio after day 31 (95% CI)	*p*‐value (interaction with time > 31 days)
**PrEP method refilled**		<.001			
Oral PrEP	91 (91, 98)		reference	reference	
Dapivirine ring	149 (132, 180)		**0.55 (0.467, 0.648)**	**0.77 (0.696, 0.858)**	<.001
**Country**		<.001			
Kenya	104 (92, 121)		**0.57 (0.467, 0.698)**	**0.77 (0.660, 0.890)**	0.021
Lesotho	62 (43, 70)		reference	reference	
South Africa	181 (149, 216)		**0.46 (0.367, 0.589)**	**0.50 (0.417, 0.600)**	0.626
Uganda	121 (98, 131)		**0.55 (0.454, 0.673)**	**0.69 (0.597, 0.801)**	0.073
Zimbabwe	121 (102, 130)		**0.61 (0.502, 0.730)**	**0.67 (0.574, 0.771)**	0.439
**Age**		<.001			
15−24 years	72 (66, 85)		**1.99 (1.738, 2.285)**	**1.77 (1.609, 1.958)**	0.178
25+ years	157 (139, 181)		reference	reference	
**Prior PrEP status at enrolment**		<.001			
PrEP naïve	82 (71, 91)		reference	reference	
Previous PrEP user	140 (123, 164)		**0.49 (0.391, 0.613)**	**0.76 (0.666, 0.872)**	<.001
Current PrEP user	277 (245, 337)		**0.23 (0.175, 0.311)**	**0.45 (0.387, 0.512)**	<.001
**Sex worker status** ^b^		<.001			
Sex worker	136 (126, 159)		**0.65 (0.548, 0.768)**	0.90 (0.807, 1.002)	0.001
Non‐sex worker	99 (92, 113)		reference	reference	
**Education**		0.005			
Up to completed primary	123 (103, 135)		reference	reference	
More than completed primary	110 (99, 121)		1.01 (0.863, 1.192)	**1.21 (1.074, 1.361)**	0.086
**Number of sexual partners in the past 3 months**		0.195			
0−1	104 (94, 120)		reference	reference	
>1	121 (110, 127)		**0.77 (0.660, 0.898)**	1.04 (0.935, 1.151)	0.002
**Current contraceptive use**		0.030			
Yes	121 (109, 124)		**0.86 (0.755, 0.990)**	0.94 (0.855, 1.041)	0.308
No	99 (91, 116)		reference	reference	
**Any condomless vaginal sex in the past month**		0.520			
No condomless sex	106 (93, 121)		reference	reference	
Any condomless sex	117 (102, 121)		0.99 (0.863, 1.142)	0.96 (0.870, 1.067)	0.732
**Worry about HIV**		0.223			
Not worried	120 (108, 121)		reference	reference	
Worried	100 (91, 117)		**1.23 (1.058, 1.433)**	0.98 (0.873, 1.100)	0.019
**Has primary partner**		0.533			
Yes	108 (98, 120)		**1.29 (1.029, 1.615)**	0.95 (0.826, 1.097)	0.026
No	127 (121, 143)		reference	reference	
**Marital status**		<.001			
Never married	101 (92, 116)		reference	reference	
Married/living as married	113 (97, 122)		0.91 (0.787, 1.055)	**0.84 (0.755, 0.937)**	0.394
Divorced/separated/widowed	136 (127, 159)		**0.77 (0.622, 0.946)**	**0.80 (0.692, 0.920)**	0.759
**Pregnancy status at enrolment**		0.224			
Pregnant	113 (91, 121)		1.15 (0.913, 1.444)	1.06 (0.896, 1.263)	0.599
Non‐pregnant	114 (103, 121)		reference	reference	
**Breastfeeding status at enrolment**		0.211			
Breastfeeding	94 (90, 119)		1.08 (0.884, 1.322)	1.08 (0.933, 1.251)	0.997
Non‐breastfeeding	119 (104, 121)		reference	reference	
**Time to facility**		0.578			
Less than half hour	119 (103, 121)		reference	reference	
Half to 1 hour	104 (93, 121)		1.14 (0.973, 1.336)	1.00 (0.889, 1.128)	0.200
More than 1 hour	121 (91, 134)		0.84 (0.646, 1.092)	1.04 (0.878, 1.226)	0.183
**Currently in school**		<.001			
Yes	78 (66, 91)		1.15 (0.946, 1.406)	**1.43 (1.239, 1.647)**	0.085
No	121 (111, 122)		reference	reference	

*Note*: Hazard ratios in bold reflect those with *p* < 0.05.

Abbreviations: CI, confidence interval; PrEP, pre‐exposure prophylaxis.

^a^ Participants who chose a method at enrolment but reported never initiating the method were excluded from this analysis.

^b^ Sex worker status was defined only for participants ages 18 years and above. Sex worker status was defined either by self‐report and/or through clinic record documentation of sex worker status.

Some significant interactions between baseline factors and time before/after day 31 on the hazard ratio (HR) of discontinuation were identified, but results were generally consistent (i.e. HR was in the same direction in both time periods). Appendix S4 presents method‐specific analyses.

Common reasons for discontinuation included logistical challenges, such as having no time/transportation for resupply or travel. Other reasons included no longer needing PrEP and side effects (Appendix S5). Notably, reasons were only known for discontinuers who were reached (49%, *n* = 1311/2674).

### 3.5 Total volume dispensed

From May 2023 through July 2024, a total of 8664 months’ supply of oral PrEP was dispensed, compared to 5013 months’ supply of dapivirine ring. On average, 3.1 months’ supply of oral PrEP and 3.9 months’ supply of ring were dispensed for each person who received that method.

### 3.6 HIV acquisitions

During the analysis period, 12 participants tested positive for HIV according to national HIV testing algorithms (1.27 acquisitions per 100 person‐years [95% CI: 0.656, 2.218]), including six who chose oral PrEP and six who chose the ring at enrolment (Appendix S6 details method‐specific rates). One additional participant who chose oral PrEP self‐reported a positive HIV test during a missed visit contact, but this result could not be confirmed, and the participant was classified as HIV indeterminant.

Of 12 confirmed seroconversions, 10 occurred without the participant having had a post‐enrolment negative HIV test result at least 3 months into the study (i.e. most acquisitions were identified at the 1‐month or first quarterly visit). In one instance, a person had a negative test at enrolment, and then did not return for testing until 10 months post‐enrolment. Therefore, undetected acute infection at the time of PrEP initiation cannot be ruled out for 10 of 12 individuals.

One post‐enrolment seroconversion occurred 8.5 months after enrolment in a participant who selected ring at enrolment and returned regularly for ring refills and HIV testing. The other post‐enrolment seroconversion occurred approximately 7 months after enrolment; the participant chose oral PrEP at enrolment, had returned regularly for PrEP refills and reported use in the 3 months prior to seroconversion.

## 4 DISCUSSION

In this large‐scale study designed to inform programmatic rollout, we demonstrate that women took advantage of PrEP choice. When offered oral PrEP and the dapivirine ring in existing public PrEP delivery programmes, women frequently reported selecting the method they perceived would be easy for them to use. We observed a relatively high rate of early discontinuation, but self‐reported adherence was relatively high for both products, and few participants switched from their initial method.

Our study shows the benefit of providing PrEP choice in HIV prevention programmes, which responds to demonstrated need [17, 18, 24]. There was continued high interest in oral PrEP, with participants preferring it due to ease of use and efficacy. Ring uptake was comparable to baseline preferences observed in the REACH study, a randomized, open‐label cross‐over trial assessing the safety and acceptability of the ring and oral PrEP among AGYW [25]. Of those preferring ring, more than half preferred it because it does not require daily pill taking and is easy to use. Ring users generally had higher refill rates, higher self‐reported adherence and greater continuation compared to oral PrEP users. Importantly, previous PrEP users had greater odds of choosing the ring and were less likely to discontinue compared to PrEP‐naïve individuals. This implies that offering multiple PrEP formulations could potentially re‐engage individuals in need of HIV prevention who may have been dissatisfied with oral PrEP [9].

In SSA, where women bear the greatest burden of HIV acquisition, offering products that women are comfortable with, such as the dapivirine ring, may result in improved and persistent PrEP use, thus improving effective HIV prevention coverage. The dapivirine ring could be a valuable alternative in situations where other methods are not an option or feasible, including where restrictions exist for specified populations, where community delivery of some PrEP formulations is challenging, or where product stock‐outs occur.

A high risk of discontinuation was observed among younger women (≤ 24 years) for both PrEP options, underscoring the need for additional support and targeted interventions to help AGYW use PrEP effectively, as detailed in multiple studies [26, 27]. In clinical trials of CAB PrEP and lenacapavir, AGYW had high product adherence, suggesting injectable formulations may be acceptable to young women and may foster effective PrEP use [28, 29]. CATALYST will introduce CAB PrEP in its second stage. As PrEP options expand to include long‐acting injectables, continued research is needed to understand the impact on uptake, use and overall prevention coverage.

Additionally, we observed that sex workers had higher odds of choosing ring in univariable analysis and a lower risk of PrEP discontinuation, although this relationship is likely impacted by potential confounders, including age. While this finding merits further research, it could be driven by high‐risk perception [30], which has been identified as a motivating factor for PrEP continuation among sex workers in SSA [31, 32]. Additionally, enhanced discretion of the ring could appeal to sex workers given potential stigma related to oral PrEP [33, 34, 35].

This study was conducted in five countries and in varied PrEP service delivery sites, which increases generalizability. However, several limitations exist. We report on interim analyses; results may change as CAB PrEP is added and with longer follow‐up time. Additionally, descriptive analyses may not have accounted for all potential confounders nor explored potentially meaningful interactions. Selection bias is also possible because individuals who are comfortable using oral PrEP may have declined enrolment, or conversely, the study may have selected women with a keen interest in using the ring as it was largely unavailable outside of CATALYST sites. Implementation of the service delivery package varied, and sites differed in terms of populations served, which could have impacted observed uptake and use patterns across sites and countries. The impact of the complete enhanced service delivery package outside of providing PrEP choice will be explored in future analyses. We observed a rate of HIV acquisition lower than those seen in comparable populations [12, 28]. It is possible that most HIV acquisitions observed in CATALYST occurred prior to PrEP initiation, but we are unable to confirm this. We could not determine the HIV status of those who did not return. We also experienced challenges reaching participants who stopped returning to sites, which limited our understanding of the reasons for discontinuation.

## 5 CONCLUSIONS

In this study delivering informed PrEP choice in public health settings in SSA, women took advantage of the available PrEP options and chose methods that were easy for them to use. Few HIV acquisitions were observed. However, additional support is required to address the high risk of early PrEP discontinuation. Evidence from this work will inform country decision‐making on the programmatic rollout of PrEP choice.

## COMPETING INTERESTS

The authors declare no conflicts of interest.

## AUTHORS’ CONTRIBUTIONS

VAF and EI led the development and writing of this manuscript. KT and EI served as protocol chairs for the study, and NPN, KK, EG, CAA and EI served as country principal investigators. PJ, IM and SM served as project advisors and provided input into the study design. RD, JK, IS, MC, MK, AM, TY, TM and CM served as research coordinators and oversaw staff training and data collection, and provided input into data analysis. Data analysis, interpretation and verification were led by DT, ME, MC, M‐TL, RS, GC, TY and AC. JR, KK and AK also provided input into data interpretation and results presentation. HS, RW, NM, PM and MD provided input into study design and implementation from their respective ministries of health. All co‐authors provided input into the manuscript and reviewed and approved the final version.

## FUNDING

This manuscript is made possible by the generous support of the American people through the U.S. President's Emergency Plan for AIDS Relief (PEPFAR) and the U.S. Agency for International Development (USAID).

## DISCLAIMER

The contents are the responsibility of the Maximizing Options to Advance Informed Choice for HIV Prevention (MOSAIC) project and do not necessarily reflect the views of PEPFAR, USAID or the U.S. Government. MOSAIC is a global cooperative agreement (Cooperative Agreement 7200AA21CA00011) led by FHI 360, with core partners Wits RHI, Pangaea Zimbabwe, LVCT Health, Jhpiego and AVAC.

## Supporting information




**Appendix S1**. Country‐specific definitions of “mature minors”
**Appendix S2**. Country‐specific PrEP product eligibility criteria
**Appendix S3**. Factors associated with refill at one month
**Appendix S4**. Method‐specific factors associated with PrEP discontinuation
**Appendix S5**. Reasons for PrEP discontinuation

## Data Availability

De‐identified data for the CATALYST study will be made publicly available following the completion of Stage II of the study through an open data platform, if possible. Data can also be requested from the study team. Further inquiries can be directed to vafonner@gmail.com.
