# Supplementary material for: PrEP choice in the real world: Results of a prospective cohort study describing uptake and use patterns of oral PrEP and the dapivirine vaginal ring among women in sub‐Saharan Africa
Source: J Int AIDS Soc. 2025 Jul 2;28(Suppl 2):e26457. doi: 10.1002/jia2.26457 (PMC12215814; doi:10.1002/jia2.26457)
Supplement: Supplementary file 1 — Appendix S1. Country‐specific definitions of “mature minors” Appendix S2. Country‐specific PrEP product eligibility criteria Appendix S3. Factors associated with refill at one month Appendix S4. Method‐specific factors associated with PrEP discontinuation Appendix S5. Reasons for PrEP discontinuation [file JIA2-28-e26457-s001.docx]

**Supplementary Appendices for “PrEP choice in the real world: Results of a prospective cohort study describing uptake and use patterns of oral PrEP and the dapivirine vaginal ring among women in sub-Saharan Africa”**

**Supplementary Appendix 1. Country-specific definitions of “mature minors”**

| **Country** | **CATALYST definition of emancipated minor** |
| --- | --- |
| Kenya | Aged15-17 years and married, has children, are head of their household, or cater for their own livelihood |
| Lesotho | Aged 15-17 years and married or who are unmarried with children |
| South Africa | Not applicable. Only participants aged 18 years and above were eligible to enroll in the study |
| Uganda | Aged 15-17 years and pregnant, married, has a child, or cater for their own livelihood |
| Zimbabwe | Aged 16-17 years and married (including customary law union where spouses are cohabiting); or head of household or caters for their own livelihood |

**Supplementary Appendix 2. Country-specific PrEP product eligibility criteria**

| **Country** | **Oral PrEP Eligibility** | **Dapivirine Ring Eligibility** |
| --- | --- | --- |
| **Kenya** | HIV negative with ongoing risk of HIV infection (defined categories); ≥15 years old; ≥30 kg; sexually active; not suspected of acute HIV infection; no risk event within last 72 hours; no contraindications; no severe renal disease (creatinine <50 ml/min); willing to take PrEP as prescribed  **PERMITTED during pregnancy & breastfeeding** | HIV negative; ≥18 years old; engage in vaginal intercourse; no contraindication to ARV-dapivirine (suspected of AHI, allergy or hypersensitivity to active substance, within 6 weeks post-partum); willing to effectively use PrEP ring as prescribed  **PERMITTED during pregnancy & breastfeeding** |
| **Lesotho** | HIV negative; ≥30 kg; sexually active and at substantial risk of acquiring HIV infection or requesting PrEP; not suspected of acute HIV infection; willing to take PrEP as prescribed; minimal risk of renal impairment  **PERMITTED during pregnancy & breastfeeding** | HIV negative; ≥18 years old; sexually active and at substantial risk of acquiring HIV infection or requesting PrEP; not suspected of acute HIV infection; willing to take PrEP as prescribed  **PERMITTED during pregnancy & breastfeeding** |
| **South Africa** | HIV negative; ≥15 years old (Tanner stage 3 and above can be applied for 14 years); ≥35 kg and tanner stage ≥3; not suspected of acute HIV infection; no risk event within last 72 hours; no contraindications ; No renal impairment  **PERMITTED during pregnancy (if serum creatinine <85 mmol/L) & breastfeeding** | HIV negative; ≥18 years old  **NOT PERMITTED during pregnancy or breastfeeding** |
| **Uganda** | HIV negative; no age restrictions (lowest age group: adolescents – 10 years); at substantial risk of HIV infection; willingness to use PrEP as prescribed.; no suspicion of acute HIV infection; no exposure to HIV in last 72 hours; no contraindications  **PERMITTED during pregnancy & breastfeeding** | HIV negative; ≥18 years old; Willingness to use ring effectively and attend scheduled follow-up visits; no suspicion of acute HIV infection; no exposure to HIV in last 72 hours; no contraindications  **NOT PERMITTED during pregnancy or breastfeeding** |
| **Zimbabwe** | Aged 18+ years OR emancipated minors between the ages 16-17; HIV-uninfected, sexually active,  and are at substantial risk of HIV acquisition; no exposure to HIV in the past 72 hours; no suspicion of acute HIV infection (AHI); No allergy to TDF and / or FTC/3TC in the PrEP regimen or other substances listed in the product information sheet; Must be willing, able, and ready to adhere to daily pill regimen and periodic HIV testing; No renal impairment. Creatinine clearance must be > 60ml/min; Belonging to a high risk population group (Sex workers, sero-discordant couple, AGYW, PBFP)  **PERMITTED during pregnancy & breastfeeding** | Aged 18+ years;  HIV-uninfected, sexually active,  and are at substantial risk of HIV acquisition; no exposure to HIV in the past 72 hours; no suspicion of AHI; No allergy to dapivirine in the PrEP ring or other substances listed in the product information sheet; Must be willing, able, and ready to adhere to use the PrEP ring consistently as prescribed and periodic HIV testing; Has a vagina (assigned female at birth).  **NOT PERMITTED during pregnancy**  **PERMITTED during breastfeeding** |

**Supplementary Appendix 3. Factors associated with refill at one month**

| **Participant characteristics ^†^** | **M1 Return^‡^ (n = 1409)** | **No Return (n = 2100)** | **Total N (n = 3509)** | **Unadjusted OR (95% CI)** | **p-value** | **Adjusted OR (95% CI)** | **p-value** |
| --- | --- | --- | --- | --- | --- | --- | --- |
| **PrEP method refilled** |  |  |  |  |  |  |  |
| Oral PrEP | 765 (32.7) | 1577 (67.3) | 2342 (66.7) | reference | _ |  |  |
| Dapivirine ring | 644 (55.2) | 523 (44.8) | 1167 (33.3) | 2.54 (2.198, 2.932) | <.001 | 2.25 (1.917, 2.634) | <.001 |
| Total | 1409 | 2100 | 3509 |  |  |  |  |
| **Country** |  |  |  |  |  |  |  |
| Kenya | 321 (44.7) | 397 (55.3) | 718 (20.5) | 1.89 (1.530, 2.337) | <.001 | 1.52 (1.200, 1.919) | <.001 |
| Lesotho | 236 (29.9) | 552 (70.1) | 788 (22.5) | reference | _ |  |  |
| South Africa | 220 (52.1) | 202 (47.9) | 422 (12) | 2.55 (1.995, 3.252) | <.001 | 2.17 (1.658, 2.848) | <.001 |
| Uganda | 276 (35.4) | 504 (64.6) | 780 (22.2) | 1.28 (1.036, 1.583) | 0.022 | 1.12 (0.890, 1.399) | 0.342 |
| Zimbabwe | 356 (44.4) | 445 (55.6) | 801 (22.8) | 1.87 (1.522, 2.300) | <.001 | 1.57 (1.252, 1.957) | <.001 |
| Total | 1409 | 2100 | 3509 |  |  |  |  |
| **Age** |  |  |  |  |  |  |  |
| 15-24 | 524 (32.2) | 1101 (67.8) | 1625 (46.3) | 0.54 (0.468, 0.617) | <.001 | 0.67 (0.570, 0.781) | <.001 |
| 25+ | 885 (47) | 999 (53) | 1884 (53.7) | reference | _ |  |  |
| Total | 1409 | 2100 | 3509 |  |  |  |  |
| **Prior PrEP status at enrollment** |  |  |  |  |  |  |  |
| PrEP Naive | 925 (36.4) | 1616 (63.6) | 2541 (72.8) | reference | _ |  |  |
| Previous PrEP User | 253 (46.9) | 286 (53.1) | 539 (15.4) | 1.55 (1.281, 1.864) | <.001 | 1.22 (0.996, 1.496) | 0.055 |
| Current PrEP User | 230 (56.1) | 180 (43.9) | 410 (11.7) | 2.23 (1.807, 2.757) | <.001 | 1.46 (1.162, 1.847) | 0.001 |
| Total | 1408 | 2082 | 3490 |  |  |  |  |
| **Sex worker status** |  |  |  |  |  |  |  |
| Sex worker | 389 (43.8) | 499 (56.2) | 888 (25.4) | 1.21 (1.039, 1.414) | 0.014 | 0.84 (0.697, 1.017) | 0.074 |
| Non sex worker | 1020 (39.1) | 1586 (60.9) | 2606 (74.6) | reference | _ |  |  |
| Total | 1409 | 2085 | 3494 |  |  |  |  |
| **Education** |  |  |  |  |  |  |  |
| Up To Completed Primary | 324 (39.9) | 489 (60.1) | 813 (23.3) | reference | _ |  |  |
| More Than Completed Primary | 1083 (40.5) | 1594 (59.5) | 2677 (76.7) | 1.03 (0.874, 1.204) | 0.759 |  |  |
| Total | 1407 | 2083 | 3490 |  |  |  |  |
| **Number of sexual partners in past 3 months** |  |  |  |  |  |  |  |
| 0-1 | 971 (40.2) | 1444 (59.8) | 2415 (69.6) | reference | _ |  |  |
| >1 | 431 (40.8) | 625 (59.2) | 1056 (30.4) | 1.03 (0.885, 1.188) | 0.737 |  |  |
| Total | 1402 | 2069 | 3471 |  |  |  |  |
| **Current contraceptive use** |  |  |  |  |  |  |  |
| Yes | 810 (42.1) | 1113 (57.9) | 1923 (55.3) | 1.20 (1.043, 1.371) | 0.010 | 0.98 (0.844, 1.133) | 0.766 |
| No | 589 (37.8) | 968 (62.2) | 1557 (44.7) | reference | _ |  |  |
| Total | 1399 | 2081 | 3480 |  |  |  |  |

^†^ There are missing data for various variables, and these observations are excluded from the respective models.

^‡^ Returned at month 1 is defined as refilled more of the same method received at enrollment within 60 days after enrollment. Subjects who switched their method after enrollment were counted as no refill. Subjects receiving more than 1 month supply at enrollment are also excluded.

**Supplementary Appendix 4. Method-specific factors associated with PrEP discontinuation^†^**

**Oral PrEP**

| **Participant characteristics ^‡^** | **Median Survival (days)** | **log rank p-value (overall)** | **Hazard Ratio before day 31 (95% CI)** | **Hazard Ratio after day 31 (95% CI))** | **p-value (interaction with time > 31 days)** |
| --- | --- | --- | --- | --- | --- |
| **Country** |  | <.001 |  |  |  |
| Kenya | 82 (66, 91) |  | **0.67 (0.528, 0.837)** | **0.82 (0.679, 0.981)** | 0.173 |
| Lesotho | 41 (36, 61) |  | reference | reference |  |
| South Africa | 186 (152, 232) |  | **0.44 (0.341, 0.577)** | **0.40 (0.322, 0.498)** | 0.561 |
| Uganda | 93 (74, 121) |  | **0.54 (0.432, 0.676)** | **0.69 (0.582, 0.822)** | 0.086 |
| Zimbabwe | 118 (95, 126) |  | **0.59 (0.477, 0.723)** | **0.57 (0.480, 0.678)** | 0.831 |
| **AGYW** |  | <.001 |  |  |  |
| Yes | 63 (45, 68) |  | **2.01 (1.721, 2.354)** | **1.86 (1.652, 2.099)** | 0.438 |
| No | 135 (125, 157) |  | reference | reference |  |
| **Prior PrEP status at enrollment** |  | <.001 |  |  |  |
| PrEP Naive | 66 (62, 71) |  | reference | reference |  |
| Previous PrEP User | 126 (107, 141) |  | **0.56 (0.425, 0.740)** | **0.78 (0.655, 0.938)** | 0.046 |
| Current PrEP User | 296 (277, .) |  | **0.20 (0.133, 0.287)** | **0.35 (0.288, 0.421)** | 0.009 |
| **Sex worker status** |  | <.001 |  |  |  |
| Sex worker | 133 (119, 150) |  | **0.70 (0.573, 0.860)** | **0.81 (0.705, 0.939)** | 0.243 |
| Non sex worker | 89 (73, 91) |  | reference | reference |  |
| **Education** |  | 0.013 |  |  |  |
| Up To Completed Primary | 103 (91, 127) |  | reference | reference |  |
| More Than Completed Primary | 91 (87, 97) |  | 1.05 (0.873, 1.269) | **1.21 (1.043, 1.396)** | 0.259 |
| **Number of sexual partners in past 3 months** |  | 0.173 |  |  |  |
| 0-1 | 91 (80, 93) |  | reference | reference |  |
| >1 | 110 (93, 125) |  | 0.85 (0.706, 1.018) | 0.98 (0.852, 1.116) | 0.228 |
| **Current contraceptive use** |  | 0.082 |  |  |  |
| Yes | 97 (91, 119) |  | 0.90 (0.777, 1.054) | 0.93 (0.827, 1.049) | 0.771 |
| No | 91 (73, 92) |  | reference | reference |  |
| **Any condomless vaginal sex in the past month** |  | 0.029 |  |  |  |
| No condomless sex | 91 (78, 95) |  | reference | reference |  |
| Any condomless sex | 93 (89, 110) |  | 1.03 (0.884, 1.210) | 0.91 (0.803, 1.027) | 0.203 |
| **Worry about HIV** |  | 0.937 |  |  |  |
| Not Worried | 92 (91, 104) |  | reference | reference |  |
| Worried | 92 (75, 103) |  | 1.12 (0.944, 1.340) | 0.94 (0.818, 1.084) | 0.122 |
| **Has primary partner** |  | 0.275 |  |  |  |
| Yes | 91 (89, 95) |  | 1.25 (0.960, 1.633) | 1.02 (0.849, 1.226) | 0.213 |
| No | 124 (102, 134) |  | reference | reference |  |
| **Marital Status** |  | <.001 |  |  |  |
| Never Married | 89 (72, 92) |  | reference | reference |  |
| Married/Living as Married | 95 (91, 111) |  | **0.85 (0.719, 0.996)** | **0.85 (0.747, 0.965)** | 0.974 |
| Divorced/Separated/Widowed | 130 (95, 152) |  | 0.78 (0.605, 1.002) | **0.77 (0.639, 0.930)** | 0.953 |
| **Pregnancy status at enrollment** |  | 0.609 |  |  |  |
| Pregnant | 113 (91, 121) |  | 0.98 (0.775, 1.250) | 0.95 (0.795, 1.145) | 0.839 |
| Non-pregnant | 91 (89, 97) |  | reference | reference |  |
| **Breastfeeding status at enrollment** |  | 0.648 |  |  |  |
| Breastfeeding | 91 (75, 107) |  | 0.98 (0.793, 1.219) | 1.06 (0.901, 1.246) | 0.588 |
| Non breastfeeding | 92 (91, 101) |  | reference | reference |  |
| **Time to facility** |  | 0.246 |  |  |  |
| Less Than Half Hour | 92 (91, 103) |  | reference | reference |  |
| Half To One Hour | 91 (68, 101) |  | **1.20 (1.006, 1.427)** | 1.01 (0.879, 1.171) | 0.149 |
| More Than One Hour | 117 (90, 132) |  | 0.80 (0.595, 1.075) | 1.00 (0.823, 1.219) | 0.213 |
| **Currently in school** |  | <.001 |  |  |  |
| Yes | 68 (63, 82) |  | 1.11 (0.887, 1.382) | **1.48 (1.254, 1.755)** | 0.039 |
| No | 97 (91, 109) |  | reference | reference |  |

^†^ Subjects who received PrEP at enrollment but then reported that they had not used it at all are excluded from this analysis. Please see below for a complete definition of discontinuation.

^‡^ There are missing data for various variables, and these observations are excluded from the respective models.

**Dapivirine Ring**

| **Participant characteristics ^‡^** | **Median Survival (days)** | **log rank p-value (overall)** | **Hazard Ratio before day 31 (95% CI)** | **Hazard Ratio after day 31 (95% CI))** | **p-value (interaction with time > 31 days)** |
| --- | --- | --- | --- | --- | --- |
| **Country** |  | 0.282 |  |  |  |
| Kenya | 148 (118, 195) |  | 0.72 (0.451, 1.146) | 0.92 (0.697, 1.218) | 0.371 |
| Lesotho | 131 (91, 163) |  | reference | reference |  |
| South Africa | 155 (125, 216) |  | 0.71 (0.401, 1.253) | 0.89 (0.633, 1.256) | 0.500 |
| Uganda | 181 (128, 231) |  | 0.87 (0.543, 1.382) | 0.79 (0.593, 1.064) | 0.758 |
| Zimbabwe | 135 (104, 190) |  | 0.90 (0.565, 1.448) | 1.02 (0.762, 1.373) | 0.664 |
| **AGYW** |  | <.001 |  |  |  |
| Yes | 119 (93, 125) |  | **1.63 (1.216, 2.175)** | **1.55 (1.304, 1.849)** | 0.789 |
| No | 197 (163, 221) |  | reference | reference |  |
| **Prior PrEP status at enrollment** |  | <.001 |  |  |  |
| PrEP Naive | 121 (104, 130) |  | reference | reference |  |
| Previous PrEP User | 180 (133, 215) |  | **0.52 (0.348, 0.771)** | 0.86 (0.691, 1.060) | 0.029 |
| Current PrEP User | 235 (200, 263) |  | **0.38 (0.242, 0.589)** | **0.68 (0.547, 0.842)** | 0.020 |
| **Sex worker status** |  | 0.656 |  |  |  |
| Sex worker | 159 (126, 195) |  | 0.73 (0.535, 1.004) | 1.15 (0.968, 1.374) | 0.014 |
| Non sex worker | 149 (129, 181) |  | reference | reference |  |
| **Education** |  | 0.340 |  |  |  |
| Up To Completed Primary | 162 (123, 211) |  | reference | reference |  |
| More Than Completed Primary | 149 (129, 180) |  | 0.82 (0.595, 1.135) | 1.20 (0.975, 1.466) | 0.054 |
| **Number of sexual partners in past 3 months** |  | 0.032 |  |  |  |
| 0-1 | 179 (136, 195) |  | reference | reference |  |
| >1 | 134 (121, 163) |  | 0.86 (0.636, 1.158) | **1.31 (1.099, 1.554)** | 0.017 |
| **Current contraceptive use** |  | 0.929 |  |  |  |
| Yes | 149 (127, 181) |  | 0.92 (0.678, 1.238) | 1.04 (0.868, 1.243) | 0.483 |
| No | 159 (128, 190) |  | reference | reference |  |
| **Any condomless vaginal sex in the past month** | 175 (143, 201) | .0333 | reference | reference |  |
| No condomless sex | 145 (126, 181) |  | 0.96 (0.702, 1.301) | 1.12 (0.936, 1.352) | 0.375 |
| Any condomless sex | 175 (143, 201) |  | reference | reference |  |
| **Worry about HIV** |  | 0.035 |  |  |  |
| Not Worried | 159 (133, 185) |  | reference | reference |  |
| Worried | 133 (99, 175) |  | **1.67 (1.227, 2.273)** | 1.06 (0.865, 1.295) | 0.015 |
| **Has primary partner** |  | 0.232 |  |  |  |
| Yes | 150 (132, 181) |  | 1.17 (0.764, 1.805) | 0.81 (0.649, 1.019) | 0.138 |
| No | 159 (120, 192) |  | reference | reference |  |
| **Marital Status** |  | 0.053 |  |  |  |
| Never Married | 134 (121, 171) |  | reference | reference |  |
| Married/Living as Married | 183 (132, 236) |  | 1.00 (0.714, 1.404) | **0.75 (0.609, 0.921)** | 0.151 |
| Divorced/Separated/Widowed | 151 (131, 209) |  | 0.92 (0.628, 1.358) | 0.87 (0.700, 1.087) | 0.800 |
| **Pregnancy status at enrollment** |  | 0.200 |  |  |  |
| Pregnant | 112 (38, 148) |  | 1.33 (0.548, 3.243) | 1.40 (0.789, 2.480) | 0.929 |
| Non-pregnant | 155 (134, 181) |  | reference | reference |  |
| **Breastfeeding status at enrollment** |  | 0.603 |  |  |  |
| Breastfeeding | 183 (92, 258) |  | 0.99 (0.537, 1.818) | 0.90 (0.627, 1.297) | 0.801 |
| Non breastfeeding | 149 (132, 179) |  | reference | reference |  |
| **Time to facility** |  | 0.686 |  |  |  |
| Less Than Half Hour | 149 (129, 183) |  | reference | reference |  |
| Half To One Hour | 163 (123, 218) |  | 0.82 (0.558, 1.210) | 0.96 (0.772, 1.182) | 0.504 |
| More Than One Hour | 132 (92, 209) |  | 0.89 (0.504, 1.576) | 1.07 (0.774, 1.469) | 0.591 |
| **Currently in school** |  | 0.055 |  |  |  |
| Yes | 121 (71, 183) |  | 1.21 (0.772, 1.885) | 1.27 (0.969, 1.661) | 0.850 |
| No | 156 (134, 183) |  | reference | reference |  |

^‡^ There are missing data for various variables, and these observations are excluded from the respective models.

**Definition of discontinuation:** Discontinuation was defined as cessation of any PrEP method at the end of the analysis period, which either occurred at the start of Stage II or date of data cut-off (July 31, 2024). Discontinuation was inclusive of gaps in PrEP use during Stage I and excluded product switching (i.e., those who switched products were not considered to have discontinued PrEP). Participants were only classified as “discontinued” if they were in a gap at the end of the analysis period, with a gap defined as either PrEP supply exhausted without refill within 30 days and without reporting use in the last 30 days since their last study contact, or until the last time PrEP use was reportedly stopped among those who did not return for another PrEP refill. Those reporting PrEP use through self-report after visits to a CATALYST site ceased were considered to have “stopped PrEP use” at their last study contact as these participants were no longer receiving PrEP in the context of choice and their continued PrEP use could not be verified. Participants reporting PrEP use in the last 30 days at the Stage II initiation visit were censored at the start of Stage II (end of Stage I).

**Supplementary Appendix 5. Reasons for PrEP discontinuation^†^**

**Oral PrEP users**

| **Reason for stopping PrEP** | **Among oral PrEP users**^‡^  **N= 917** |
| --- | --- |
| No time / transport for resupply | 357 (38.9%) |
| Ran out of pills/expired | 220 (24.0%) |
| No longer needed | 159 (17.3%) |
| Traveling / far away | 107 (11.7%) |
| Disliked side effects | 78 (8.5%) |

^†^Participants could select more than one reason for stopping PrEP.

^‡^ Defined as having used oral PrEP as last method prior to PrEP discontinuation among those who were reached following a missed visit contact or clinic visit and provided a response for reason for stopping PrEP.

**Dapivirine ring users**

| **Reason for stopping PrEP** | **Among dapivirine ring users^§^**  **N=394** |
| --- | --- |
| No time / transport for resupply | 152 (38.6%) |
| Traveling / far away | 80 (20.3%) |
| Ran out / expired | 71 (18.0%) |
| No longer needed | 47 (11.9%) |
| Disliked side effects | 25 (6.4%) |

^§^Defined as having used the dapivirine ring as last method prior to PrEP discontinuation among those who were reached following a missed visit contact or clinic visit and provided a response for reason for stopping PrEP.

**Supplementary Appendix 6. HIV acquisition rates**

| **PrEP choice at enrollment** | **n** | **Total person year at risk** | **Number of incident acquisitions** | **Incidence Rate (95% CI)** |
| --- | --- | --- | --- | --- |
| **Overall** | 3967 | 945.1225 | 12^†^ | 1.27 (0.656, 2.218) |
| Oral PrEP | 2627 | 546.0917 | 6 | 1.1 (0.403, 2.391) |
| Dapivirine Ring | 1187 | 388.1615 | 6 | 1.55 (0.567, 3.364) |
| No Method | 148 | 10.8693 | 0 | 0 (0, 33.939) |
| Unknown | 5 | 0 | 0 | _ |

^†^ Ten of twelve of incidence HIV acquisitions occurred without the participant having had a post-enrolment negative HIV-test result at least three months into the study (i.e., most infections acquisitions were identified at the month one or first quarterly visit). Therefore, undetected acute infection at the time of PrEP initiation cannot be ruled out for 10 of 12 individuals.
